# The Washout Resistance of Bioactive Root-End Filling Materials

**DOI:** 10.3390/ma16175757

**Published:** 2023-08-23

**Authors:** Joanna Falkowska, Tomasz Chady, Włodzimierz Dura, Agnieszka Droździk, Małgorzata Tomasik, Ewa Marek, Krzysztof Safranow, Mariusz Lipski

**Affiliations:** 1 Department of Preclinical Conservative Dentistry and Preclinical Endodontics, Pomeranian Medical University in Szczecin, Al. Powstanców Wlkp. 72, 70-111 Szczecin, Poland; wlodzimierz.dura@pum.edu.pl (W.D.); ewa.marek@pum.edu.pl (E.M.); 2 Faculty of Electrical Engineering, West Pomeranian University of Technology in Szczecin, Sikorsky 37 St., 70-313 Szczecin, Poland; tchady@zut.edu.pl; 3 Department of Interdisciplinary Dentistry, Pomeranian Medical University in Szczecin, Al. Powstanców Wlkp. 72, 70-111 Szczecin, Poland; agnieszka.drozdzik@pum.edu.pl (A.D.); malgorzata.tomasik@pum.edu.pl (M.T.); 4 Department of Biochemistry and Medical Chemistry, Pomeranian Medical University, Al. Powstanców Wlkp. 72, 70-111 Szczecin, Poland; krzysztof.safranow@pum.edu.pl

**Keywords:** bioactive cements, washout resistance, retrograde root canal filling

## Abstract

Fast-setting bioactive cements were developed for the convenience of retrograde fillings during endodontic microsurgery. This in vitro study aimed to investigate the effect of irrigation on the washout of relatively fast-setting materials (Biodentine, EndoCem Zr, and MTA HP) in comparison with MTA Angelus White and IRM in an apicectomy model. Washout resistance was assessed using artificial root ends. A total of 150 samples (30 for each material) were tested. All samples were photographed using a microscope, and half of them were also scanned. The samples were irrigated and immersed in saline for 15 min. Then the models were evaluated. Rinsing and immersing the samples immediately after root-end filling and after 3 min did not disintegrate the fillings made of all tested materials except Biodentine. Root-end fillings made of Biodentine suffered significant damage both when rinsing was performed immediately and 3 min after the filling. Quantitative assessment of washed material resulted in a slight loss of IRM, EndoCem MTA Zr, and MTA HP. MTA Angelus White showed a slightly greater washout. Rinsing and immersion of Biodentine restorations resulted in their significant destruction. Under the conditions of the current study, the evaluated materials, excluding Biodentine, showed good or relatively good washout resistance.

## 1. Introduction

Surgical endodontic treatment is performed when orthograde root canal therapy is unsuccessful and retreatment is either impossible or useless [1]. Endodontic surgery usually involves exposure of the apex, the removal of pathological periapical tissue, root-end resection (apicoectomy), root-end cavity preparation, and the placement of a retrograde filling material [1]. The success rate of this surgical procedure is above 90% and is close to that of orthograde root canal treatment [2,3,4].

Several materials have been used in periapical surgery, including amalgam, intermediate restorative material (IRM), super ethoxybenzoic acid (Super-EBA), glass ionomer cements, polycarboxylate cements, zinc phosphate cements, calcium phosphate cements, composite resins, and calcium silicate cements, but none of them have the characteristics of an ideal root-end filling material [5,6,7,8,9]. An ideal root-end filling material should provide a long-term hermetic seal resulting from resistance to washout and dissolution in periapical fluids and good adaptation to the walls of retrograde preparation. It must be non-irritating, non-toxic, non-carcinogenic, and biocompatible. For clinical applications, it should be easy to manipulate and radiopaque [1,5,7,8].

Mineral trioxide aggregate (MTA) is usually used as the material of choice for retrograde root canal filling because it has most of the properties of an ideal root-end filling material, such as bioactivity and biocompatibility, antimicrobial effect, good sealing ability, and setting in a humid environment [1,7,8,9,10,11,12,13,14,15]. Many authors also claim that these materials evoke a positive tissue response to promote the regeneration of the periodontium [16,17,18]. However, MTA has some drawbacks, such as a long setting time, difficult application, low resistance to compression and flexion, and a high cost [6,19,20,21,22]. Many studies have also reported that MTA leads to tooth discoloration [23,24,25].

In order to improve the clinical results of the treatment, many new calcium silicate materials have been developed. Biodentine^®^ (Septodont, Saint Maur des Fossés, France), a new dental substitute, was introduced on the market in 2009. Biodentine is composed of a powder component (mainly calcium silicates) and a liquid component (water with calcium chloride and a water-soluble polymer). The powder is placed in a capsule, while the liquid is in an ampoule. Mixing is achieved using a trituator for 30 s at 4000–4200 rpm. According to the manufacturer, the initial setting time is about 12–15 min [26]. However, some authors [27] estimated the final setting time of this material to be 85 min. The consistencies of Biodentine and phosphoric cement are similar, which makes Biodentine easy to apply [28]. As for MTA, the indications and clinical applications for Biodentine are indirect/direct pulp capping, pulpotomy, apexogenesis, apexification (apical plug), root and crown perforation, resorption repair, and retrograde root-end filling [28,29,30]. Because Biodentine was released at the end of 2009, many laboratory studies have been published so far with this material. The literature is still, however, inconclusive concerning the superiority of Biodentine over MTA as a root-end filling material in apical surgery. In some reports, Biodentine produced a better seal than MTA, whereas other studies failed to confirm any superiority of Biodentine or found it to be inferior to MTA when filling the retrograde cavities. A recently published meta-analysis showed that there is a lack of scientific evidence for the superiority of Biodentine over MTA as a retrograde filling material in apical surgery [31].

Both MTA (e.g., ProRoot MTA, Dentsply Sirona, Charlotte, NC, USA, MTA Angelus, Angelus, Londrina, Brazil) and Biodentine are calcium silicate cements. In the process of cement setting, hydration is a very important reaction. The main product of this reaction is calcium silicate hydrate. Some authors conduct research on the effect of the presence of aluminum on the effectiveness of this reaction. Although the presence of aluminum may affect the efficiency of the reaction, it may also decrease its biocompatibility and lengthen the setting time. Among tested materials, aluminum is present in MTA-type materials, but fully synthetic Biodentine does not contain it [31,32].

Another newly developed type of bioactive material for end filling is EndoCem Zr (Maruchi, Wonju, Republic of Korea). It has been introduced as an MTA-derived pozzolan cement (a naturally occurring siliceous and aluminous material of volcanic origin) [33]. EndoCem Zr has a short setting time of 4 min and favorable manipulation properties [33,34]. Pozzolan, when mixed with water, undergoes a reaction with calcium hydroxide to form calcium silicate hydrate, similar to that produced by the hydration of MTA. EndoCem/EndoCem Zr sets fast despite no accelerator. The faster setting of the material is probably due to the small particles of cement and thus a larger contact surface with water [35,36]. It has low tooth tissue discoloration potential because it contains zirconium oxide instead of conventional bismuth oxide [34]. However, an in vitro study showed that Endocem Zr was more cytotoxic and associated with lower expression of VEGF and ANG in comparison with mineral trioxide aggregate [37], which was confirmed by histopathologic analysis in a canine model of pulpotomy that showed fewer odontoblast layer formation and fewer calcific barrier formation with greater inflammatory response in comparison with the mineral trioxide aggregate [38].

A new type of MTA, MTA repair high plasticity (MTA HP, Angelus, Londrina, Brazil), has been introduced recently. It is based on the standard MTA formula but contains calcium tungstate (CaWO_4_) as a radiopacifier and a liquid consisting of water and a plasticizing agent [39,40]. This new composition retains the chemical properties of the original MTA but amends its physical properties (plasticity), making it easier to manipulate and insert into the retrograde cavity than traditional MTA [40,41]. MTA HP compared to ProRoot MTA sets faster (12 min) [39]. The more rapid setting of this calcium silicate cement is explained in the literature by the large surface area of the powder particles and the absence of sulphate phases [42].

One of the characteristics of the ideal material for a retrograde root canal filling is resistance to washout, i.e., the resistance of freshly prepared cement to disintegration upon early contact with fluid [43]. The term washout originated in engineering science and is used to describe washing out the material from freshly mixed cement with water [44]. In dentistry, the washout may have a considerable impact on the result of the treatment because the loss of the filling material may be the reason for microleakage. That is one of the reasons why washout resistance has already been studied by other researchers over the years [45,46]. After filling the root-end preparation, it is recommended that the resection cavity be rinsed gently; bleeding that occurs during periapical surgery is also responsible for the disintegration of the filling [47]. Therefore, the cements used for the retrograde filling should be resistant to washout. This resistance increases with time, although it should be borne in mind that it is challenging to guarantee dryness during surgery for a few minutes.

This in vitro study aimed to investigate the effect of irrigation on washout of relatively fast-setting materials (Biodentine, EndoCem Zr, and MTA HP) in comparison with root-end filling materials that have been on the market for several years, MTA Angelus White, and IRM, in an apicectomy model.

## 2. Materials and Methods

The following materials were used in this study:

1. Intermediate Restorative Material (IRM; Dentsply Sirona, Charlotte, NC, USA);
2. MTA Angelus White (Angelus, Londrina, Brazil);
3. Biodentine (Septodont, Saint Maur-des-Fossés, Cedex, France);
4. EndoCem Zr (Maruchi, Wonju, Republic of Korea);
5. MTA HP (Angelus, Londrina, Brazil).

Their compositions are outlined in Table 1.

IRM, MTA Angelus White, EndoCem Zr, and MTA HP powder were mixed manually with their liquid according to recommendations from the manufacturer. Biodentine was mixed in the trituator (Silver MIX, GC Dental, Tokyo, Japan) for 30 s.

### 2.1. Assessment of Washout Resistance

**Figure 1:**
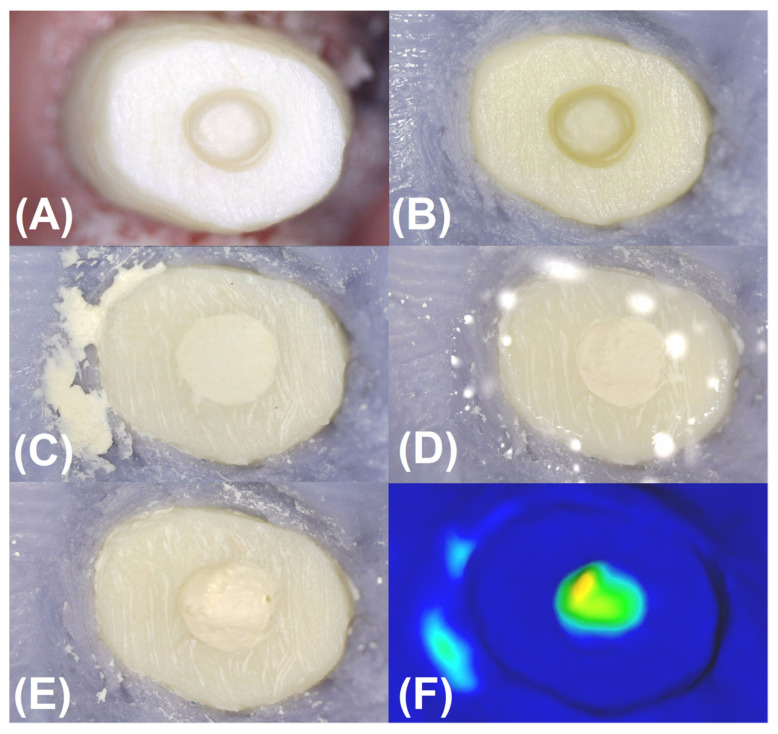
(**A**) The crown of the mandibular incisor (plastic block) after preparation to resemble the resected root with a root-end preparation of 1.2 mm × 3 mm (diameter and depth). (**B**) The bottom of the bony crypts were simulated with the use of silicone impression material. (**C**) Root-end preparation filled with retrograde material. (**D**) Rinsing of the resected root surface and the artificial bony crypt. (**E**). Partial rinsing of the material from the root-end preparation. (**F**) The color model in the form of a depth map obtained as a result of imposing scans.

Washout resistance was assessed using artificial root-end preparations of 1.2 mm × 3 mm (diameter and depth corresponding to the diameter and height of the cylinder) in plastic blocks. For this purpose, the crowns of incisors placed in the model of the mandible (Frasaco, Tettnang, Germany) were prepared to resemble resected roots (Figure 1A), and the bottom of the bony crypts were simulated with the use of silicone impression material (Figure 1B). In total, 150 samples (30 for each material) of freshly prepared materials were placed in artificial root-ends prepared in plastic blocks (Figure 1C). The application time for each material was 30 s. Materials were placed in the plastic blocks by using the MTA+ Applicator (Cerkamed, Stalowa Wola, Poland; https://cerkamed.pl/produkt/aplikator-mta/ accessed on 20 August 2023) and condensed with retro filing plugger No. 1 (Medesy SRL, Maniago, Italy; www.medesy.it/en/products/endodontic-instrument-with-plugger/ accessed on 20 August 2023).

#### 2.1.1. Experiment 1

Seventy-five samples (15 of each material) were photographed using a Levenhuk DTX 90 microscope (Levenhuk, Inc., Tampa, FL, USA) at 60× magnification. The resolution of the microscope, experimentally determined using a microscopic slide with a micrometer scale, was 10 µm. In addition, several fillings were evaluated by watching the material-wall interface using microscopes with a resolution better than 10 µm (Digital Microscope VHX-7000, Keyence, Osaka, Japan, and Digital Micro Hardness Tester, Model: MHVD—1000IS, INNOVATEST, Wiry, Poland). It was found that the width of the narrowest gaps recorded with the use of the microscope used in the present study ranged from 7–8 to 20–25 µm. If the quality of the filling was questionable, the test sample was replaced with a new one. The photography of the sample (samples were prepared one by one and photographed one by one) was taken immediately after the material application was finished (the time of taking the photography was max. 60 s) and the rinsing started immediately. For this purpose, 5 mL saline (ambient temperature) was applied for 15 s using a disposable syringe and needle of 0.8 mm diameter and 12 mm length. The saline from the needle opening impinged on the edge as in the clinical setting, i.e., the saline flowed over the resected surface, washing over the test material but not directly spraying into it (Figure 1D). After the simulation of the rinsing of the bony crypt, the models were immersed in warm saline (34 °C) for 15 min (simulation of blood flooding the crypt). After this time, the models were removed, gently dried without disrupting the fillings, and then photographed again under a microscope (Figure 1E).

#### 2.1.2. Experiment 2

The remaining 75 samples (15 of each material) were used in this experiment. Apart from photographing the samples under a microscope, the surface of the fillings was additionally scanned using KaVo ARCTICA AutoScan—the 3D dental scanner (KaVo, Biberach, Germany). Scanning and photography lasted 3 min, which can be assumed to be the time of protection against humidity in a clinical setting. Then, the samples were flushed in the same way as in experiment 1 and immersed in warm saline for 15 min, after which time the models were removed, photographed under the microscope, and scanned again using a 3D dental scanner (KaVo ARCTICA AutoScan, KaVo, Biberach, Germany). Then, the recorded scans were superimposed to obtain a color model in the form of a depth map (Figure 1F).

### 2.2. Qualitative Analysis of the Marginal Adaptation of the Materials to the Walls of the Root-End Preparations and Disintegration of Fillings

The adaptation of the retrograde materials to the walls of root-end preparations and the disintegration of root-end fillings were evaluated based on photographs. A total of 150 coded photographs from samples in groups 1–10 were evaluated by three evaluators who had been calibrated before the assessment. Each evaluator gave an independent score without reference to the other evaluators.

**Figure 2:**
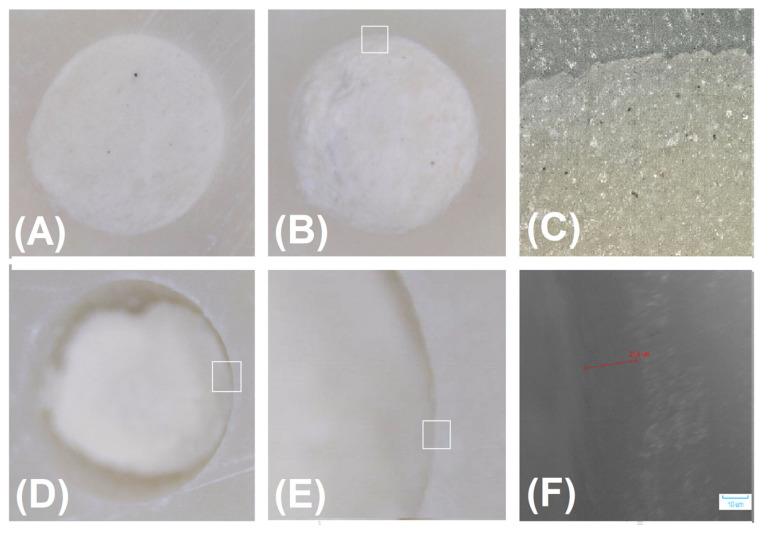
Criteria for marginal adaptation of retrograde material to the root-end cavities and washed-out area: (**A**) Score 1: Close marginal approximation of the filling material to the wall of preparation, no gaps present at the material-wall interface, no washed-out area (hollow area) on the surface of the material; (**B**) Score 2: No gaps present at the material-wall interface, washed-out areas (hollow areas) on the surface of the material. (**C**). View of the rectangular in (**B**)—close marginal approximation of the filling material to the wall of preparation (photography taken at 500× using Digital Microscope VHX-7000, Keyence, Osaka, Japan), (**D**): Score 3: Presence of narrow gaps at the material-wall interface, washed-out area (hollow area) on the surface of the material. (**E**) Extensive washout of the material. (**F**) View of the rectangular in (**E**)—the gap between the material and the wall preparation is 21 µm wide (photography taken at 500× using Digital Micro Hardness Tester, Model: MHVD—1000IS, INNOVATEST, Wiry, Poland).

The qualitative analysis of the marginal adaptation of retrograde material to the root-end cavities and washed-out area was based on the following grading criteria (Figure 2):

Score 1: Close marginal approximation of the filling material to the wall of preparation, no gaps present at the material-wall interface, no washed-out area (hollow area) on the material’s surface.

Score 2: No gaps present at the material-wall interface, washed-out area (hollow area) on the material’s surface.

Score 3: The presence of gaps at the material-wall interface and the washed out area (hollow area) on the material’s surface.

Those criteria were created based on the sample evaluation by Tran et al. [48].

### 2.3. Quantitative Assessment of the Volume of Washed out Material

**Figure 3:**
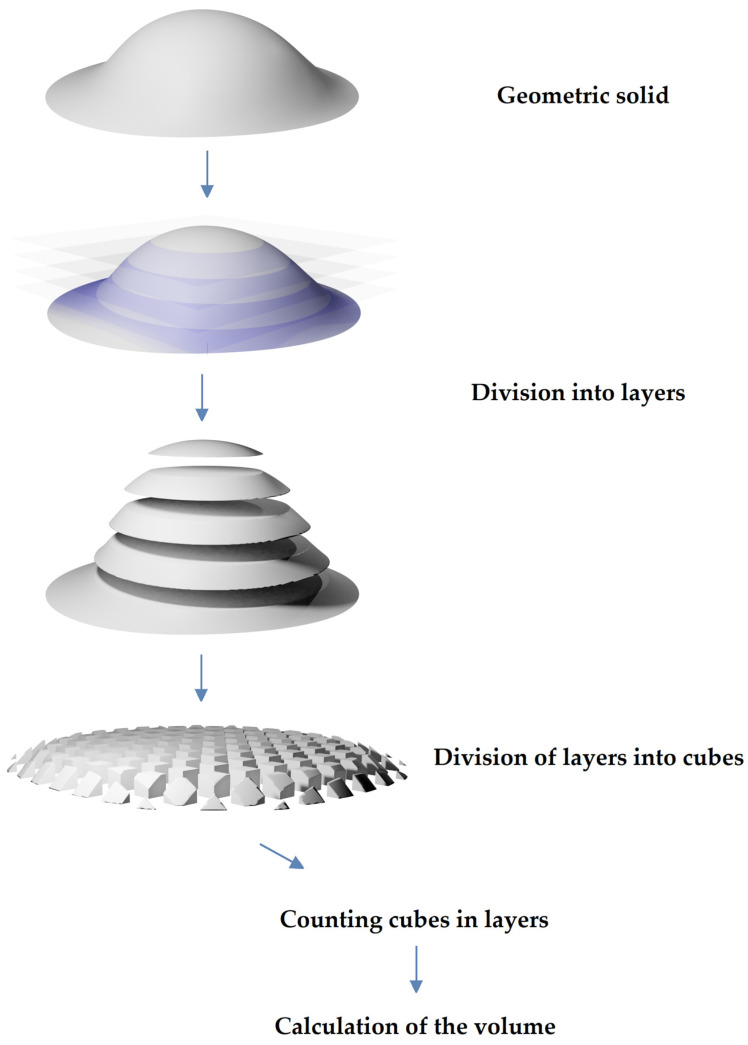
Schematic drawing of quantitative estimation of the volume of the geometric solid.

The software written by the authors in the programming language and numeric computing environment Matlab (The MathWorks, Inc., Torrance, CA, USA) was used for the quantitative assessment of the volume of washed-out material. The washed-out material was quantified by superimposing the scans recorded before and after rinsing. In this way, a geometric solid was created in the form of a depth map (a two-dimensional table with the depths of the cavity at a given point). The geometric solid was divided into layers (within a single solid, the layers had the same thickness, e.g., 5.1282 µm; layer thickness within individual solids ranged from 4 to 7 µm) and the layers into cuboids (within a single solid, the width and depth of cuboids were always the same, e.g., 4.486 × 4.486 µm^2^; within individual solids, the width and depth of cuboids ranged from 4 to 7 µm, the height of cuboids depended on the thickness of the layer, e.g., 5.1282 µm) of equal volume. Then the number of cuboids was counted, and the result was multiplied by the volume of the cuboid. In this way, the approximate volume of the solid was determined. The volume is given in mm^3^ and as a % of the filling (a cylinder with a diameter of 1.2 mm and a height of 3 mm). In addition, the average value in the depth map table was calculated, which is the average depth of the hollow (Figure 3).

### 2.4. Statistical Analysis

The mean score of qualitative analyses by three evaluators was compared between materials or between experiments with a non-parametric Kruskal–Wallis test or Mann–Whitney test. A weighted kappa was used as a measure of the evaluators’ assessment concordance. Quantitative washout values were presented as means with a standard deviation (SD). They were compared between materials with ANOVA followed by Tukey’s post hoc test after log transformation to obtain the homoscedasticity (*p* > 0.05, Levene test) and normality of distributions (*p* > 0.1, Shapiro–Wilk test) necessary for parametric analysis. A result was considered statistically significant at *p* < 0.05. Statistica 13 was used for statistical analyses.

## 3. Results

### 3.1. Qualitative Analysis of the Marginal Adaptation of the Materials to the Walls of the-End Preparations and Disintegration of Fillings

**Figure 4:**
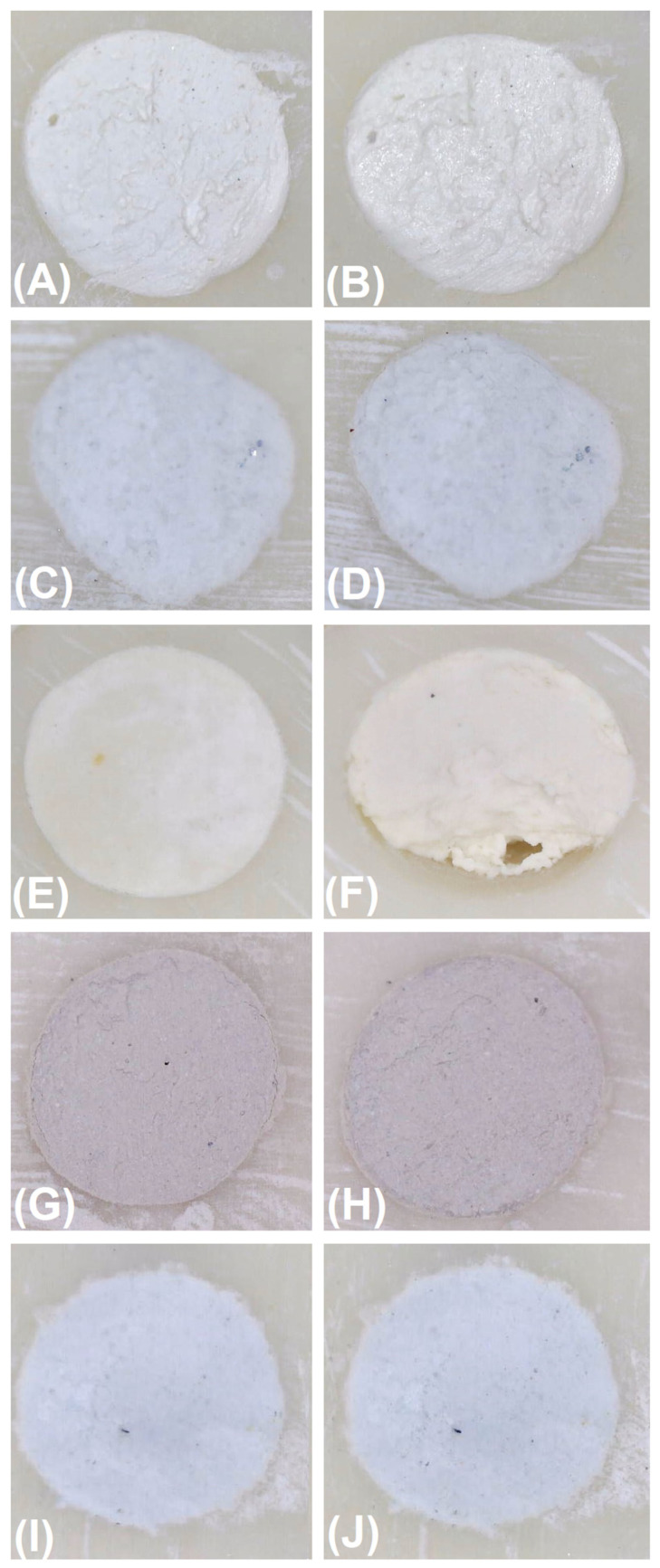
Adaption of retrograde materials to the walls of root-end preparations and disintegration of root-end filling. (**A**)—IRM before and (**B**) after rinsing and immersion of the samples in simulated blood; (**C**) MTA Angelus White before and (**D**) after rinsing and immersion of samples in simulated blood; (**E**) Biodentine before and (**F**) after rinsing and immersing the samples in simulated blood; (**G**) EndoCem MTA Zr before and (**H**) after rinsing and immersion of samples in simulated blood; (**I**) MTA HP before and (**J**) after rinsing and immersion of samples in simulated blood.

**Figure 5:**
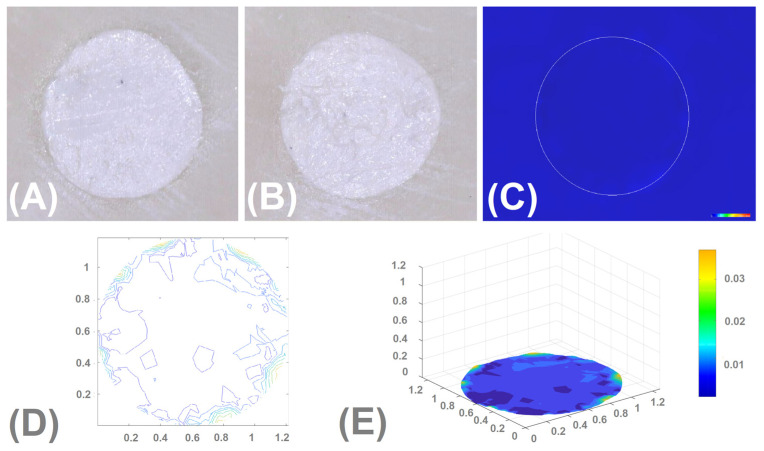
IRM. (**A**)—the image taken before rinsing and immersion in solution; (**B**)—the image taken after rinsing and immersion of the sample in solution—filling adheres tightly to the walls, visible loss of material indicating the washout; (**C**)—depth map of the cavity (washed out material); (**D**)—isolines showing the depth of the cavity/loss; (**E**)—3D chart of the depth of the cavity/loss.

**Figure 6:**
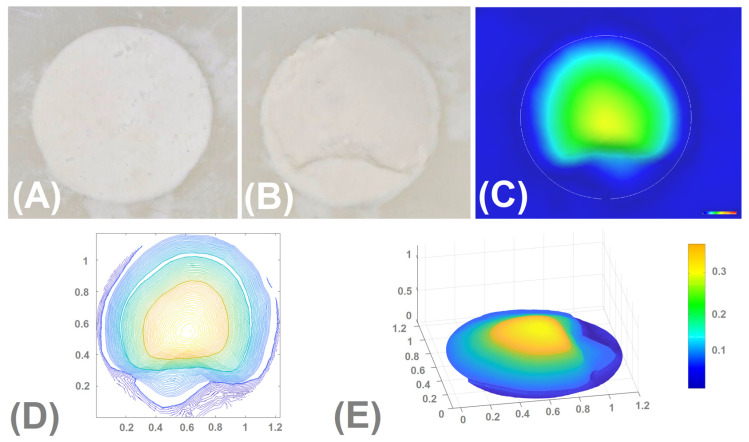
Biodentine. (**A**)—the image taken before rinsing and immersion in solution; (**B**)—the image taken after rinsing and immersion of the sample in the solution—filling adheres tightly to the walls, visible loss of material indicating the washout; (**C**)—depth map of the cavity (washed out material); (**D**)—isolines showing the depth of the cavity/loss; (**E**)—3D chart of the depth of the cavity/loss.

Rinsing and immersion of the samples in the liquid simulated blood immediately after root-end filling did not disintegrate the fillings made of IRM, MTA Angelus White, EndoCem MTA Zr, and MTA HP (Table 2). No disintegration of the fillings made of these materials occurred when the rinsing procedure was performed 3 min after the application of the materials into the root-end cavities (Figure 2, Figure 3, Figure 4 and Figure 5). On the other hand, root-end fillings made of Biodentine suffered significant damage both when rinsing was performed immediately after the application of materials into the root-end cavity and 3 min after filling (Table 2, Figure 6). There was no significant difference in the degree of damage depending on the time from application to rinsing. The weighted Cohen’s kappa statistic showed very good agreement between the three evaluators (Table 3).

### 3.2. Quantitative Assessment of Washed out Material

Rinsing and immersion of the samples in a simulated blood solution resulted in a slight loss of IRM, EndoCem MTA Zr, and MTA HP. A slightly greater washout was observed with restorations made of MTA Angelus White. However, rinsing and immersion of Biodentine restorations in a simulated blood solution resulted in significant destruction. When these volumes were translated into depth changes, the mean depths of the cavity/loss within restorations made of the studied materials ranged from 0.0132 (IRM) to 0.2230 mm Biodentine). Statistical analysis showed statistically significant differences between MTA Angelus White and other materials and between Biodentine and other materials (Table 4).

Figure 5 and Figure 6 show the images captured by the microscope, a depth map of the cavity (washed out material), isolines showing the depth of the cavity/loss, and a 3D chart of the depth of the cavity/loss regarding the IRM (the lowest volumetric changes) and Biodentine (the highest volumetric changes).

## 4. Discussion

One of the desirable features of hydraulic-setting materials placed at the root end during periapical surgery is washout resistance. Therefore, newly introduced materials on the market should not show a tendency to disintegrate as a result of contact with irritants used for rinsing the resection cavity or with body fluids [5,6,7,8,47,49].

The current study aimed to compare the washout of relatively new calcium silicate cements to that of commonly used retrograde root canal materials: MTA Angels White and IRM. In order to assess the degree of washout, a model very similar to the clinical situation was used.

Visual assessment of the degree of washout of the materials rinsed immediately after placement in the retrograde cavities and 3 min after the completion of filling showed spacing defects at the material-wall interface and hollow areas on the surface of the material, essentially only in the case of the Biodentine. This confirms previous studies evaluating resistance to washout using the basket drop, a method that gives quantitative evidence of the amount of material lost when subjected to tissue fluids and irrigating solutions during the placement of root-end materials [49]. In the cited study, Biodentine washed out to a significant extent (50% of fill weight), while with radiopacified TCS cement, Bioaggregatte and IRM washed out slightly (below 10% of fill weight). The authors of the cited study explain such a high washout by the presence of a soluble polymer in the liquid, which contributed to the reduction of the water–cement ratio without affecting the workability of the resultant cement mix. In this way, it is possible to reduce the volume of water needed to mix the material and thus improve the strength properties of the cement. On the other hand, a water-soluble polymer has the effect of a surface-active agent and thus will scatter the cement particles by applying a charge to their surfaces. This scattering will lead to a fluid mixture, which results in the dislodgement of Biodentine when tested for washout [49].

In our own study, the microscopic assessment of the washout of materials showed no deterioration of marginal integrity or formation of a hollow area in the cases of EndoCem MTA, MTA HP, IRM, and Angelus MTA White. While the high resistance to washout of IRM and EndoCem MTA according to visual assessment is not surprising as this property has been demonstrated in previous studies [50], the fairly good washout resistance found in MTA Angelus White was unexpected. Partial or complete leaching of MTA was most often reported in the literature [47,50,51,52]. The above differences should be explained primarily by differences in the research methodology and perhaps by the fact that the currently produced MTA Angelus White differs in physical and chemical properties from the MTA Angelus White used years ago. The material currently available on the market sets within 12 min, and the one produced years ago sets within 40 min (the initial setting time). However, it is known that the tendency to wash out the material increases with the setting time of hydraulic calcium silicate cements [47]. However, to the best of our knowledge, there is no information in the literature regarding the washout resistance of the MTA HP material.

In the present study, two experiments were performed to evaluate the washout resistance of the retrograde filling materials. In the first experiment, the time between application and rinsing was 1 min (the time necessary to take a picture in the microscope); in the second experiment, a 3-min interval was used (the time necessary to take the picture and scan the surface of the restorations). Thanks to this, in the second experiment, it was possible to assess the degree of washout of the material not only visually (qualitatively) but also quantitatively. By superimposing the scan of the fillings taken before rinsing on the scan of the fillings taken after rinsing, the volume of the washed material was obtained. Quantification of the washed material confirmed the poor resistance to washout of the Biodentine as observed visually. The quantitative examination also showed a slight washout of MTA Angelus White, which was not observed visually. This observation suggests that visual evaluation allows the estimation of the washed material in cases of significant material loss. This observation suggests that visual assessment allows for the estimation of washed material in cases of significant loss of material. However, if the loss of material is insignificant, it is impossible to visually assess the loss of material under a light microscope. More precise observations can be made with a scanning microscope, which allows the depth of field yield to be assessed. Unfortunately, conventional SEM allows viewing the preparation only once (it is not possible to compare the surface of the sample before and after rinsing, as the sample is destroyed during the first test). Although environmental SEM (ESEM) or low-voltage mode of SEM operation are available where the sample can be viewed more than once (with this type of microscope/operation mode, it is not necessary to cover the viewed sample with a layer of conductive material, so it is hypothetically possible to rinse it and view it again), they cannot be used due to the time-consuming nature of the test (the sample will set before the test is performed) [53,54]. In the present study, quantitative evaluation of the washout of Endocem MTA and IRM showed minimal loss of material (within error), which is consistent with previous observations [50].

Quantitative assessment of the washed material has so far been done by mass loss. For this purpose, the material was injected into distilled water for 24 h. After this time, the sample was dried, and after comparison with the initial mass, the percentage weight loss of cement [52] was determined. Some authors placed the evaluated material in bovine serum beakers and shook them. The samples were removed from the shaker for evaluation after being shaken for 0, 5, 10, 30, and 60 min, and the percentage weight loss was assessed [43].

Another objective method of washout resistance, based on the assessment of mass loss, was described by Formosa et al. [47]. These authors adopted the test method used to estimate the resistance of freshly prepared cement to washing out in the water. This original method involves placing the studied cement into a perforated vessel, allowing it to sink freely through the water, and then raising it back up. The test cycle is repeated several times, and the mass of material washed following each cycle is estimated. The main difference between the original method and the modified one lies in the size of the samples of the studied material and the size of the device constructed for dental testing (both samples and the measuring device are correspondingly smaller) and in immersing them not in tap water but in distilled water and/or HBSS.

In the present study, a quantitative evaluation of the washed material was made by comparing the scan of the surface of the fillings registered immediately after their placement with the scan registered after rinsing and immersion in solution. A similar methodology has so far been used only by Smith et al., who used a profilometer instead of a scanner [55]. In the cited study, however, the washout was not determined, but the solubility of set calcium silicate cement in endodontic solutions (EDTA, BioPur MTAD). Fully set cements have been found to be resistant to endodontic irrigants.

The method that allows for a very accurate estimation of the lost volume of material is micro-computer tomography (micro-CT) [56]. This test allows not only the surface loss of the material but also the presence of the canal wall—retrograde filling gaps and voids within the retrograde fillings—to be assessed [57]. Micro-CT imaging is a non-invasive, highly accurate tool that has been increasingly used for the 3-dimensional assessment of microstructures. A certain limitation of the method is that only radiopaque material can be evaluated; however, the materials used for retrograde filling of the prepared canal should be radiopaque, so this is not a problem [58]. However, a significant disadvantage of this method is its time-consuming nature, which does not allow it to be used to assess washout. The scanning procedure takes so long that the material is set and, at most, its solubility but not its washout can be determined [58,59,60].

Many authors draw attention to the poor washout resistance of conventional MTA preparations. However, our own study did not show that MTA Angelus White was significantly washed out, which is to some extent confirmed by the good results of clinical trials [61,62,63,64]. Regarding Biodentine as a material for retrograde root canal filling, there are no randomized and prospective clinical trials in the available literature, although this material has been commercially available for 14 years. Only case reports and case series, which have limited scientific value, have been published in the literature [65,66,67,68].

The strength of the experiment was reproducibility; the authors did not have to limit themselves to a certain number of trials and could ensure the accuracy of the results. However, a weakness of the study was the relatively long time of the 3D scanning, which could not be reduced. Using a 3D scanner that can scan faster might be useful in future studies.

## 5. Conclusions

Under the conditions of the current study, the evaluated materials, excluding Biodentine, showed good or relatively good washout resistance. The Biodentine material was washed out both 1 and 3 min after filling, which is worrying and requires further research.

## Figures and Tables

**Table 1:** Composition of the commercial materials.

Material	Manufacturer	Ingredient	Mixing
IRM	Dentsply Sirona, Charlotte, NC, USA	powder: zinc oxide, poly-methyl methacrylate (PMMA) powder, pigment liquid: eugenol, acetic acid	1 spoon of powder + 1 drop of distilled water (mixed manually on glass slab using a metal spatula, 30 s)
MTA Angelus White	Angelus, Londrina, Brazil	powder: tricalcium silicate, dicalcium silicate, tricalcium aluminate, ferroaluminate tricalcium, calcium oxide, bismuth oxide liquid: distilled water	2 level scoops of powder + 3 drops of liquid (mixed manually on glass slab using a metal spatula, 30 s)
Biodentine	Septodont, Saint-Maur-des-Fossés Cedex, France	powder: tricalcium silicate, dicalcium silicate, calcium carbonate and oxide filler, iron oxide shade, and zirconium oxide liquid: calcium chloride as an accelerator, hydrosoluble polymer water-reducing agent, water	0.7 g capsule of powder + 5 drops of liquid (mixed in the trituator; 30 s; 4000–4200 rpm)
EndoCem Zr	Maruchi, Wonju, Republic of Korea	powder: calcium oxide, silicon dioxide, aluminum oxide, magnesium oxide, ferrous oxide, zirconium oxide liquid: distilled water	0.3 g of powder + 0.12 mL (mixed manually on glass slab using a metal spatula; 30 s)
MTA HP	Angelus, Londrina, Brazil	powder: tricalcium silicate, dicalcium silicate, tricalcium aluminate, calcium oxide, and calcium tungstate liquid: water and plasticizer	0.085 g capsule of powder + 2 drops of liquid (mixed manually on a glass slab using a metal spatula; 30 s)

**Table 2:** Distribution of scores (percentages) pooled from three examiners for each material.

Experiment	Material	Score, *n* (%)		
		1	2	3
1	IRM ^a^	45 (100)	-	-
	EndoCem Zr ^a^	44 (97.78)	1 (2.22)	-
	MTA HP ^a^	43 (95.56)	2 (4.44)	-
	MTA Angelus White ^a^	41 (91.11)	4 (8.88)	-
	Biodentine ^b^	-	13 (28.89)	32 (71.11)
2	IRM ^a^	45 (100)	-	-
	EndoCem ZR ^a^	45 (100)	-	-
	MTA HP ^a^	44 (97.78)	1 (2.22)	-
	MTA Angelus White ^a^	44 (97.78)	1 (2.22)	-
	Biodentine ^b^	-	8 (17.78)	37 (82.22)

Different letters indicate significant differences between materials (a vs. b: *p* < 0.0001 for both experiments, Kruskal-Wallis test for mean score of the evaluators). No significant differences were found between materials indicated by a (*p* > 0.5 for both experiments). There were no significant differences between experiments 1 and 2 for any of the studied materials (*p* > 0.3, Mann-Whitney test).

**Table 3:** Interexaminer differences of score for all materials pooled.

Experiment	Comparisons	Weighted Kappa	Agreement
1	Evaluator 1 vs. 2	0.861	Very good
	Evaluator 1 vs. 3	0.890	Very good
	Evaluator 2 vs. 3	0.898	Very good
2	Evaluator 1 vs. 2	0.924	Very good
	Evaluator 1 vs. 3	0.922	Very good
	Evaluator 2 vs. 3	0.922	Very good

**Table 4:** The results of the quantitative assessment of washed-out materials.

Material	Washout		
	Mean Volumetric Change ± SD (in mm^3^)	Mean Volumetric Change ± SD (in %)	Mean Depth ± SD (in mm)
IRM	0.0149 ^a^ ± 0.0021	0.4392 ^a^ ± 0.0605	0.0132 ^a^ ± 0.0018
EndoCem Zr	0.0180 ^a^ ± 0.0009	0.5300 ^a^ ± 0.0271	0.0159 ^a^ ± 0.0008
MTA HP	0.0185 ^a^ ± 0.0029	0.5442 ^a^ ± 0.0885	0.0163 ^a^ ± 0.0026
MTA Angelus	0.0305 ^b^ ± 0.0089	0.9004 ^b^ ± 0.2627	0.0270 ^b^ ± 0.0079
Biodentine	0.2521 ^c^ ± 0.0338	7.4332 ^c^ ± 0.9967	0.2230 ^c^ ± 0.0299

Different letters indicate significant differences (*p* < 0.05, ANOVA followed by Tukey’s post hoc test for log-transformed values) between materials (b vs. a: *p* < 0.005; c vs. a and c vs. b: *p* = 0.00013; no significant differences were found between three materials denoted by “a”), so the ranking of materials from best performance down is a > b > c (IRM, EndoCem Zr, MTA HP > MTA Angelus > Biodentine).

## Data Availability

Not applicable.
